# Neuron Loss in Alzheimer’s Disease: Translation in Transgenic Mouse Models

**DOI:** 10.3390/ijms21218144

**Published:** 2020-10-30

**Authors:** Oliver Wirths, Silvia Zampar

**Affiliations:** Department of Psychiatry and Psychotherapy, University Medical Center (UMG), Georg-August-University, D-37075 Göttingen, Germany; silvia.zampar@med.uni-goettingen.de

**Keywords:** Alzheimer’s disease, amyloid β, neuron loss, transgenic mice, Amyloid precursor protein, intraneuronal Aβ

## Abstract

Transgenic mouse models represent an essential tool for the exploration of Alzheimer’s disease (AD) pathological mechanisms and the development of novel treatments, which at present provide only symptomatic and transient effects. While a variety of mouse models successfully reflects the main neuropathological hallmarks of AD, such as extracellular amyloid-β (Aβ) deposits, intracellular accumulation of Tau protein, the development of micro- and astrogliosis, as well as behavioral deficits, substantial neuron loss, as a key feature of the disease, seems to be more difficult to achieve. In this review, we summarize information on classic and more recent transgenic mouse models for AD, focusing in particular on loss of pyramidal, inter-, and cholinergic neurons. Although the cause of neuron loss in AD is still a matter of scientific debate, it seems to be linked to intraneuronal Aβ accumulation in several transgenic mouse models, especially in pyramidal neurons.

## 1. Introduction

**Figure 1:**
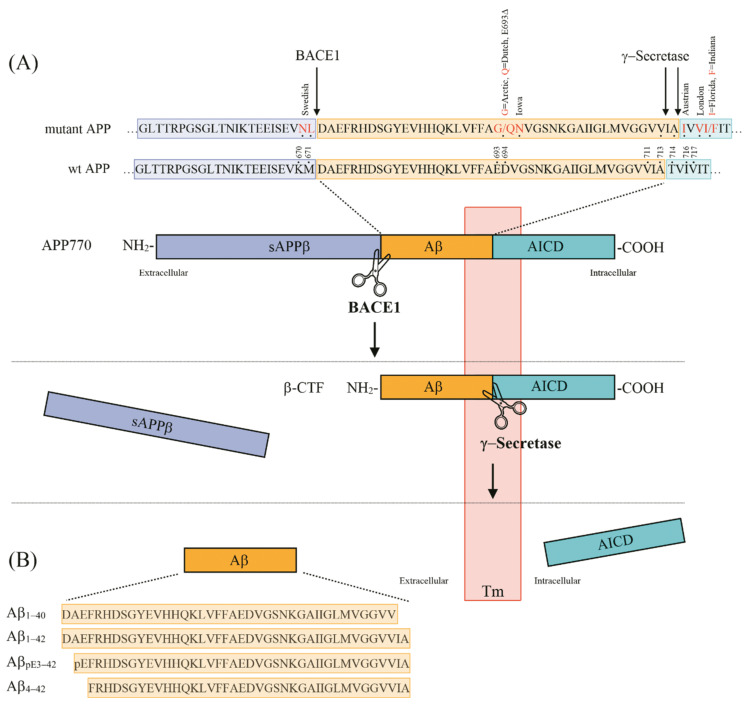
(**A**) Schematic representation of mutant and wildtype (WT) human amyloid precursor protein (APP), highlighting the positions of mutations frequently used in APP transgenic mouse models of AD (modified from [7]). Cleavage by β-secretase (BACE1) initiates the generation of Aβ peptides with the liberation of the soluble sAPPβ fragment. The membrane-bound APP C-terminal fragment (β-CTF) is further cleaved by the γ-secretase complex to liberate Aβ and the APP intracellular domain (AICD). (**B**) Amino acid sequences and numbering of the most common Aβ variants present in transgenic AD mouse models.

Alzheimer’s disease (AD) is a neurodegenerative disorder representing the most common form of dementia worldwide. It manifests clinically with progressive memory loss, cognitive decline, as well as with changes in personality and mood. Essential neuropathological hallmarks important for the diagnosis of AD consist of the extracellular deposition of amyloid-β (Aβ) peptides in plaques [1] and intracellular aggregation of hyperphosphorylated Tau protein in neurofibrillary tangles (NFTs) [2]. Aβ peptides are produced by sequential cleavage of the type-I transmembrane amyloid precursor protein (APP) by β-site APP cleaving enzyme 1 (BACE1) [3,4] and the γ-secretase complex [5], in what is referred to as the amyloidogenic APP processing pathway (Figure 1). The initial cleavage by BACE1 leads to the extracellular release of a soluble fragment called sAPPβ, leaving a membrane-bound β-C-terminal fragment (β-CTF), which is further cleaved by γ-secretase within the transmembrane domain. Full-length Aβ peptides consisting of 40 (Aβ_1–40_) or 42 (Aβ_1–42_) amino acids, as well as the remaining APP intracellular domains (AICD) are formed in this way [6].

Genetic mutations in the *APP* gene or presenilin-1 and presenilin-2 (*PS1*, *PS2*) genes, which encode for proteins constituting essential parts of the γ-secretase complex [8], were found in early onset cases of familial AD (FAD) and are responsible for an enhanced Aβ production and deposition in plaques [9]. These observations supported the amyloid cascade hypothesis and the central role of fibrillary Aβ aggregates in the etiology of the disease [10]. Even though extracellular amyloid plaque deposits had long been considered the principal players in the development and progression of AD, this association has been challenged by a poor correlation with the disease status [11] and therefore other options, such as the potential toxic effects of intraneuronal Aβ accumulation, have been considered [12]. Intraneuronal deposits of Aβ are found especially in early AD and Down syndrome brains [13,14], and might precede and enhance the formation of extracellular plaques [15,16]. Beyond full-length Aβ_1–40_ and Aβ_1–42_ species, a variety of *N*- or *C*-terminally truncated Aβ peptide variants have been identified in the human brain, presenting with different aggregation and toxicity profiles (reviewed in [17]).

Neuron loss is a basic and fundamental feature in the pathogenesis of AD [18,19], starting already at preclinical stages when the neuropathological hallmarks are not yet present [20]. A decrease in neuron numbers can be observed in different brain regions in AD patients [21], culminating in an involvement of the entire brain at late stages. In particular, loss of neurons in the CA1 region of the hippocampus [22,23] and in the entorhinal cortex seems to correlate with the severity of memory deficits [11]. Even though the exact mechanisms of neuronal death in AD are not yet fully understood, Aβ has been suggested as an initiator by interaction with and disruption of the endoplasmic reticulum (ER) and mitochondrial integrity [24] but also as a potential trigger of apoptosis [25]. In addition to a direct involvement of Aβ in pathways resulting in neurodegeneration and synaptic deficits, the peptide is believed to enhance the phosphorylation of Tau proteins that consequently act as mediators in events contributing to synaptic dysfunction and neuronal death [26]. Another important factor might be the disruption of fast axonal transport, resulting in focal swellings filled with various organelles, as seen in the vicinity but also distant from plaques in several AD transgenic models [27,28,29,30], resembling Wallerian degeneration [31]. The role of extracellular Aβ plaques is also not entirely clear: While in early studies, a substantial relationship between plaque numbers and cognitive decline in the aging population has been assumed [32], subsequent studies challenged this observation, reporting no association [33] and allocating more importance to synapse loss [34] and neurofibrillary tangle formation [35]. Nonetheless, thioflavin S-positive fibrillary Aβ deposits contribute to focal neuronal toxicity, at least in their immediate environment, as shown in both AD cases and AD transgenic mice [36] and local oxidative stress surrounding plaques has been proposed as a potential mechanism contributing to selective neuronal death [37].

In the past three decades, numerous transgenic AD mouse models have been developed with the intention to better understand the underlying disease mechanisms. The majority of the currently available models overexpress mutated forms of human APP with or without the co-expression of PS and/or Tau transgenes. Additionally, murine models expressing exclusively different variants of the Aβ sequence have been generated. Although the main neuropathological AD hallmarks as well as the behavioral impairments are largely reflected in the majority of these models to a certain extent, neuron loss remains an underrepresented manifestation that is mainly observed when intraneuronal Aβ accumulation is also present.

The generation of appropriate preclinical models is essential for the identification of disease mechanisms as well as the development of potential treatments. Since a decrease in neuron numbers is a critical component of the disease development, we report on some of the most important and widely used AD transgenic mouse models with β-amyloid pathology and provide an overview with a focus on the modelling of neuronal loss (summary in Table 1).

## 2. Intraneuronal Aβ Is Linked to Pyramidal Neuron Loss in Transgenic AD Mouse Models

### 2.1. Pyramidal Neuron Loss in Transgenic Mice with APP Overexpression

While the main neuropathological hallmarks characterizing AD, such as extracellular amyloid deposition, micro- and astrogliosis, as well as learning and memory deficits, have been successfully modelled in transgenic mice relying on *APP* overexpression, robust neuron loss is much less evident [66,67]. During the last 25 years, a multitude of different transgenic mouse lines modelling AD were developed that can be categorized according to their transgenes. Many models overexpress human mutant *APP* in the form of single transgenic mice (such as PDAPP [68], APP/Ld [69], Tg2576 [38], TgCRND8 [39], APP23 [40], tgAPP_ArcSwe_ [70], APP-Au [71], or APP_E693Δ_ [42]). In addition, several bigenic lines expressing combinations of *APP* and *PS1*/*PS2* (e.g., 5XFAD [46], APPswe/PS1dE9 [72], APP/PS1 [49], PS2APP [73], APP/PS1KI [52]), *APP* and *Tau* (such as APP/Tau [55]), or triple transgenic mice expressing *APP*, *PS1/2*, and *Tau* (e.g., 3xTg [74], 5XFAD/PS19 [56], or TauPS2APP [75]) have been generated. While most of these models present abundant extracellular amyloid plaque pathology and associated inflammatory changes, modelling significant neuron loss remained often less or only partially successful.

In recent years, a variety of studies indicated that intraneuronal Aβ accumulation is an early pathological feature in the human AD brain [12,13] that might precede and contribute to amyloid plaque pathology [15,16].

The APP23 model expressing human *APP* with the Swedish mutation under the control of the murine neuron-specific Thy1-promoter is one of the earliest models showing evidence for neuron loss. A reduction in the CA1 neuron number of 14% has been demonstrated; however, in this study, both hemi- and homozygous mice with an age of 14 to 18 months were included, which complicates interpretation of the data [41]. Surprisingly, an increased number of ~14% of neurons was reported at 8 months of age in the neocortex, while in 27-month-old animals, there was no difference compared to wildtype (WT) controls, but an inverse correlation between amyloid load and neuron number was observed [76]. A more recent study confirmed the reduced number of neurons in the hippocampal CA1 subfield [77] and early intraneuronal Aβ accumulation was reported at least in cortical layer V neurons preceding extracellular amyloid plaque formation [78].

Intraneuronal accumulation of Aβ peptides preceding plaque formation has been reported in double transgenic APP_751SL_/PS1_M146L_ mice. This mouse line accumulates intraneuronal Aβ peptides in the CA1 region of the hippocampus, as well as in the neocortex [51]. Ultrastructural analyses revealed that Aβ peptides localize subcellularly to multivesicular bodies containing lysosomal enzymes [79], confirming previous observations in the human AD brain [80]. In 17-month-old APP_751SL_/PS1_M146L_ mice, a loss of CA1–3 neurons of ~30% compared to age-matched PS1 control animals was demonstrated by unbiased stereological counting methods. Interestingly, the level of hippocampal pyramidal cell loss exceeded the amount explainable by the pure presence of extracellular plaque deposition by approximately 10%, indicating the involvement of more than one mechanism in hippocampal neuron loss in this mouse model [50]. In good agreement, an age-related loss of synaptophysin-immunoreactice presynaptic boutons, even in regions devoid of extracellular plaques, has been observed in the hippocampus of these mice [81].

**Figure 2:**
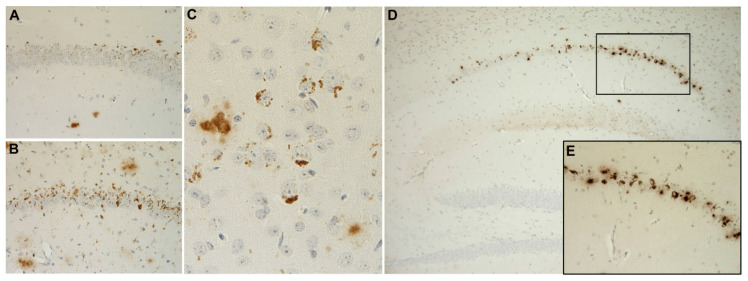
Intraneuronal Aβ accumulation in the CA1 region of 2- (**A**) and 6-month-old (**B**) APP/PS1KI mice, as well as in cortical layers ((**C**), 2-month-old). Six-month-old homozygous Tg4-42 mouse showing CA1 neuron loss together with intraneuronal Aβ accumulation (**D**,**E**) (higher magnification of area indicated in (**D**)).

Instead of human *PS1* overexpression, the same APP_751SL_ mice were crossed on a mutant *PS1* knock-in background (KI), which resulted in an aggravated plaque pathology and an earlier and more robust neurodegenerative phenotype. These APP/PS1KI mice present with early intraneuronal Aβ accumulations in the CA1 region of the hippocampus, as well as in deep cortical layers (Figure 2), and stereological quantifications revealed a ~50% loss of CA1 neurons at 10 months of age [52]. More thorough follow-up studies demonstrated a loss of ~30% of CA1 neurons already in 6-month-old animals, coinciding with a strong intraneuronal accumulation of full-length, but also N-terminally truncated, pyroglutamate-modified, as well as oligomeric Aβ species [82]. As mentioned previously, intraneuronal Aβ accumulation in this model is not restricted to the hippocampus but is also evident in deep cortical layers. A detailed quantitative analysis of neuronal numbers in the frontal cortex and thalamus revealed an early loss of cortical neurons starting at the age of 6 months. While the frontal cortex represents an area with both intra- and extracellular Aβ accumulation, no intracellular deposits are present in the thalamus. The amount of extracellular plaque pathology, however, is comparable between both brain regions, pointing to a critical role of transient intraneuronal Aβ accumulations with regard to neurodegeneration [53]. As mentioned above, focal amyloid plaque toxicity also exists to a certain degree [36] and might account for reduced granule cell numbers in the dentate gyrus, a brain region without clear evidence for intraneuronal Aβ accumulation in this mouse model [83].

The widely used 5xFAD mouse model is another transgenic line in which neuron loss has been quantitatively assessed [46]. These mice show an early onset of amyloid plaque deposition (~2 months of age) and present with neuron loss in cortical layer 5 and subiculum [46]. Interestingly, intraneuronal Aβ accumulations precede extracellular plaque deposition also in 5xFAD mice. Stereological quantifications confirmed a significant loss of layer 5 cortical neurons at 9 and 12 months of age [47,48], which is essentially prevented upon *BACE1* gene deletion [84]. At 12 months of age, no reductions in neuron numbers were detected in the CA1 layer, which, despite considerable APP expression, is devoid of intraneuronal Aβ immunoreactivity. This observation adds further evidence to the assumption that the intracellular localization of the peptide is closely associated with neurotoxicity [48]. However, it has to be noted that another study reported a loss of pyramidal neurons in the Ammon’s horn of the hippocampus, which is rescued upon deletion of endogenous murine Tau [85].

In this respect, it is interesting that a mutation identified in Japanese pedigrees with AD [86] seems to be associated with the intracellular accumulation of oligomeric Aβ peptide species. While fibrillar Aβ species have been considered to be the main neurotoxic species in former times, more recent studies suggest that pre-fibrillar small soluble oligomeric Aβ variants are at least equally toxic [87,88].

In transfected human embryonic kidney (HEK)293 cells, the introduction of the APP_E693Δ_ (“Osaka”) mutation resulted in increased β- and γ-secretase cleavage of mutant APP and promoted intracellular Aβ accumulation via decreased total Aβ secretion [89]. A transgenic mouse model overexpressing APP_E693Δ_ showed intraneuronal accumulation of oligomeric Aβ peptide species starting at the age of 8 months; however, up to the age of 24 months, no extracellular plaque deposition became evident. Interestingly, an abundant activation of micro- and astroglia together with a significant decrease in the number of NeuN-positive cells in the hippocampal CA3 region was reported at 24 months of age [42]. More thorough analyses in 22-month-old mice located the Aβ oligomers to endoplasmic reticulum (ER), endosomes/lysosomes, and mitochondria, suggesting that intraneuronal Aβ peptides cause cell death via an induction of ER stress, endosomal/lysosomal leakage, and mitochondrial dysfunction [90].

In other commonly used models, such as Tg2576 or TgCRND8, the potential relationship between intraneuronal Aβ and hippocampal neuron loss is less obvious. While in the latter model a decreased volume and CA1 neuron number has been reported [91], there is so far no direct confirmation for Aβ accumulation within neurons in this particular brain area. However, it has to be noted that although there is no clear evidence for Aβ accumulation within cell bodies, intraneuronal Aβ has been detected in axons projecting from CA1 in organotypic hippocampal slice cultures derived from TgCRND8 mice [92]. The same holds true for the Tg2576 mouse model. While no report directly links intraneuronal Aβ accumulation and neurodegeneration in these mice, a significant decrease in the thickness of the CA1 pyramidal cell layer has been demonstrated by Nissl staining [93]. Although reported mainly in cortical layer 5 neurons, analyses by immunoelectron microscopy revealed an accumulation of Aβ_42_ within multivesicular bodies in Tg2576 mice prior to the onset of extracellular plaque pathology [80].

### 2.2. Neuron Loss in Transgenic Mice with Aβ Peptide Overexpression

Over and above the overexpression of full-length *APP*, shorter transgenic constructs comprising only the Aβ sequence have also been frequently used to achieve human Aβ expression/accumulation. The initial report with evidence for neurotoxic in vivo properties of Aβ originates from a transgenic mouse model employing the mouse neurofilament light gene (NF_L_) promoter to drive neuronal Aβ expression (NF_L_-Aβ). While evidence for extracellular Aβ deposition was scarce, neuronal Aβ immunoreactivity was detected in the cortex and hippocampus, resulting in abundant neurodegeneration, with biochemical and morphological evidence for an apoptotic mechanism [61]. More recently, a transgenic line expressing Aβ_1–42_ linked to a proenkephalin signal peptide (APP48) has been described [62]. These mice present with motor impairment and reduced forebrain and spinal cord volume. They do not develop extracellular plaques; however, Aβ-positive dendrites and somatic granules were found throughout the grey matter and considerable neuron loss was detected in the CA1 layer of the hippocampus at 3 and 18 months of age [62].

In addition to full-length Aβ peptides (Aβ_1–40_, Aβ_1–42_), a variety of shorter N-terminally truncated Aβ peptides with comparable neurotoxicity have been described in recent years (reviewed in [17,94]). In the human brain, Aβ peptides starting with a pyroglutamate residue at position 3 (Aβ_pE3–x_) have been demonstrated to be present in similar or even increased amounts compared to their full-length counterparts [95]. These species are interesting because of their distinct biophysical properties, comprising enhanced aggregation propensity [96] and increased stability to resist proteolytic degradation [97]. To investigate the role of Aβ_pE3_ in vivo, transgenic lines under the control of the murine Thy1-promotor facilitating neuronal expression have been generated [63,64,98]. As pyroglutamate residues cannot be expressed directly, constructs utilizing an N-terminal glutamine (Q) residue with an appropriate signal peptide to ensure proper secretion have been employed. Pyroglutamate formation is catalyzed by the enzyme glutaminyl cyclase; however, it occurs faster from glutamine (Q) compared to glutamate (E) residues [99]. In contrast to lines based on *APP* overexpression, which usually produce a large heterogeneity of different peptides, the role of Aβ_pE_ peptides can be exclusively analyzed in these models. Expression of this Aβ_3Q–42_ construct in the TBA2 line resulted in abundant Aβ_pE_ formation in the hippocampus, as well as the Purkinje cell layer of the cerebellum. At ~2 months of age, a severe neurological phenotype with growth retardation, loss of motor coordination, and ataxia became evident, together with Aβ_pE_ accumulation and loss of Purkinje cells [63].

The TBA2.1 mouse line, expressing the same construct in a homozygous fashion, showed a related phenotype. These mice accumulated Aβ and Aβ_pE3_ mainly in the CA1 layer of the hippocampus and present with ~30% neuron loss at 3 and ~50% less neurons at 5 months of age in this brain area. This was accompanied by a robust activation of astrocytes and microglia, arguing for an Aβ_pE3_-induced cellular neurotoxicity. These mice further showed a rapid onset of motor deficits, together with tremor, rigor, and abnormal posture by 3 months of age [98].

These findings are corroborated in TBA42 mice, another transgenic line employing the Aβ_3Q–42_ construct. Intraneuronal Aβ accumulation was evident by 3 months of age in the CA1 pyramidal layer and an altered anxiety phenotype detectable already at this time point. Motor deficits and reduced performance in a spontaneous alternation task were detected at 12 months of age [64]. A more thorough behavioral analysis confirmed the severe motor phenotype and revealed additional deficits in spatial reference memory in the Morris water maze, as well as an ~35% loss of CA1 pyramidal neurons in aged TBA42 mice [100].

Aβ42 peptides starting with phenylalanine (F) at position 4 (Aβ_4–42_) are among the first Aβ peptides that have been isolated from amyloid plaque cores in the human AD brain [101] and represent a highly abundant species in brains from both sporadic and familial AD cases [102]. A transgenic mouse model (Tg4-42) expressing only Aβ4-42 under the control of the neuron-specific Thy1-promoter has been developed as a tool to assess the in vivo toxicity of this peptide. In good agreement with the abovementioned results in models expressing only specific Aβ peptide variants, these mice do not develop overt extracellular amyloid pathology [65] but robust intraneuronal Aβ accumulation, mainly in the hippocampus (Figure 2). They develop deficits in spatial reference and object recognition memory, correlating with robust neuron loss in the CA1 pyramidal layer in a gene dose-dependent manner [65,103,104], as well as motor deficits [105] and reduced neurogenesis [106]. A combination of Tg4-42 and TBA42 mice exacerbates CA1 neuron loss and motor deficits, together with a strong accumulation of Aβ peptides in motor neurons of the spinal cord [107].

## 3. Tau Triggers Neuron Loss in Models with Aβ Pathology

While transgenic models expressing human Tau are beyond the scope of the current review, several models with combined APP and Tau expression have been reported. The 3xTg mouse model expressing mutant APP and PS1, as well as Tau, has also been shown to accumulate considerable levels of intraneuronal Aβ in neocortical and hippocampal neurons starting at 3 to 4 months of age and preceding the deposition of extracellular Aβ peptides [74]. Extracellular plaque formation starts at ~6 months of age [74]; however, a much later onset at ~15 months in male mice, together with a lack of Aβ_1–42_ immunoreactivity in the CA1 pyramidal layer has also been reported [108]. Interestingly, staining with Fluoro Jade C, a marker for degenerating neurons, did not reveal evidence for neurodegeneration [109] and a quantitative analysis of CA1 neuron numbers revealed a lack of neuron loss in this brain region in 3xTg mice during aging [110]. This discrepancy to the abovementioned data in models with intraneuronal Aβ accumulation might be due to the use of APP cross-reacting antibodies (such as 6E10 or 4G8), which have been frequently used for Aβ labelling [111]. As these antibodies detect central Aβ epitopes, they usually also pick-up considerable levels of full-length APP, resulting in an overestimation of the effective intraneuronal Aβ levels in the hippocampus in this model [112].

Accelerated plaque formation together with neurofibrillary degeneration and neuronal loss has been described in Tg2576 mice crossed to the VLW lines expressing human 4-repeat Tau containing a triple mutation [55]. While neither neurofibrillary tangle formation nor significant neuron loss in CA1 has been reported in the single-transgenic Tg2576 model [66], stereology-based neuron counts revealed significantly reduced neuron numbers in the CA1 and entorhinal cortex in APP/Tau mice [55].

Crossing the 5xFAD model with PS19 mice overexpressing human mutant Tau P301S driven by the mouse prion protein promoter [113] resulted in a clear aggravation of Tau pathology, while β-amyloid pathology remained unaffected [56,57]. Co-expression of Tau lead to deficits in basal synaptic transmission, as well as spatial memory deficits [57] and increased neuroinflammation [56]. Of note, 5xFAD/PS19 mice presented with robust hippocampal atrophy and severe loss of neurons in the hippocampal CA1 layer [56,57]. These findings are corroborated in 5xFAD/Tg30 mice, a model co-expressing mutant Tau with the G272V and P301S mutations. While no difference in the number of pyramidal neurons in the hippocampus (CA1–CA4) was detected in either 9-month-old WT, 5xFAD, or Tg30 mice, a loss of ~25% of pyramidal neurons in this area was detected in age-matched 5xFAD/Tg30 mice [60].

These observations support the idea that Aβ and Tau act together and that Aβ is upstream of Tau [114,115], positioning Aβ as the initiator and Tau as the executor in the pathogenetic process [116]. In good agreement, motor and working memory deficits are rescued in 5xFAD mice lacking endogenous Tau (5xFAD/Tau^−/−^), as well as neuron numbers in the Ammon’s horn of the hippocampus [85].

## 4. Loss of Other Neuronal Cell Types in APP Transgenic Mice

### 4.1. Loss of Interneurons in Transgenic Mouse Models Expressing Mutant APP

Beyond pyramidal neurons, hippocampal network activity is determined and coordinated by inhibitory interneurons expressing the neurotransmitter γ-amino-butyric acid (GABA). These cells are often classified according to their immunoreactivity against calcium-binding proteins, such as parvalbumin (PV) or calretinin (CR) [117]. In the hippocampus of AD patients, a ~60% decrease of PV-positive interneurons has been detected in the dentate gyrus and CA1–CA2 subfields [118] and related findings were reported in the perirhinal cortex [119]. The expression of these markers seems to correlate with differential vulnerability in AD, as in the entorhinal cortex, a clear atrophy of PV-positive cells was reported [120], while in the piriform cortex, a preferential vulnerability of somatostatin- and CR-positive cells co-localizing with amyloid deposits was evident [121].

Quantitative studies investigating interneuron populations in transgenic AD mouse models yielded more conclusive results, with most of the available studies reporting a loss of CR- and PV-positive interneurons in the hippocampal formation in AD transgenic mice [59,122,123,124,125,126].

In 5xFAD mice, significantly reduced PV-positive neuron numbers were shown in cingulate and motor cortices [127], as well as cells in the cortical layer IV [128] or the hippocampal CA1–3 and dentate gyrus subfields [126]. A related finding was reported in APP/PS1KI mice, which presented with significantly reduced CR- and PV-positive neuron numbers in the hippocampus [125]. Interestingly, no changes were detected in layers V and VI of the frontal cortex in this model, although pyramidal neurons were significantly reduced and massive thioflavin S-positive extracellular amyloid deposits were present [129]. In TgCRND8 mice, a model expressing human mutant APP with the Swedish and Indiana mutations and an early onset of plaque deposition at 3 months of age [39], significantly reduced numbers of PV-positive interneurons in the CA1/2 layer were detected at only 1 month of age, a time point preceding extracellular plaque deposition [130]. Curiously, at 6 months of age, a loss of mainly neuropeptide Y-positive cells was reported in different hippocampal subfields, while PV-positive interneurons were largely unaltered [122]. In 3xTg mice, ~33% of CR- and ~52% of PV-positive cells were lost in the CA1 layer in 18-month-old animals [59]. One possible mechanism contributing to, in particular, loss of PV-positive interneurons could be the concomitant loss of other cell types providing excitatory input, such as pyramidal cells. This might result in a cascade finally causing PV-positive neuron death, proposed as a more general mechanism in neurodegenerative diseases [131]. However, the observation of hippocampal interneuron loss in models which are almost devoid of CA1 pyramidal neuron loss, such as 3xTg or 5xFAD, might be more supportive of a link between loss of interneurons and the appearance of extracellular Aβ deposits [123].

### 4.2. Cholinergic Neuron Loss in APP Transgenic Mice

Degeneration of cholinergic neurons is known to significantly contribute to cognitive decline in AD patients [132] and has been studied in a variety of genetic AD mouse models. While an age-related reduction in the density of cholinergic nerve terminals was evident in 4-month-old homozygous PDAPP mice, no difference in the number of cholinergic basal forebrain neurons was detected in even 24-month-old animals [133]. A related observation was reported in aged APP23 mice. Although cholinergic fiber length was significantly reduced in the neocortex of aged APP23 mice, no loss of cholinergic basal forebrain neurons was evident [134]; however, others described cholinergic neuron degeneration in the medial septal nucleus at 12–14 months of age [135]. On the other hand, a significant decrease in the number of cholinergic neurons was reported in 12-month-old TgSweDI mice [44], a model overexpressing APP with the Swedish, Dutch, and Iowa mutations (Tg-SwDI) under the control of the murine Thy1-promoter, which is characterized by an early extracellular plaque onset and prominent perivascular/vascular Aβ deposition [43]. Quantifications of neurons stained with choline acetyltransferase (ChAT) in the nucleus basalis of Meynert (nbM) revealed a significant decrease compared to age-matched WT animals, together with a reduction in cholinergic fiber density [44].

In good agreement, 6-month-old mice overexpressing APP with the Swedish and London mutations revealed an ~60% decrease in the number of ChAT-positive cells in the nbM [45] and reduced ChAT-positive neuron numbers were also reported in the medial septum of 3xTg [58] and 5xFAD mice [136]. The latter model also shows cholinergic neuron loss in the basal forebrain starting at 9 months of age [137]. In ChAT-positive motor neuron nuclei of APP/PS1KI mice, co-expression of the APP transgene, together with robust intracellular Aβ immunoreactivity, was detected. Stereological analyses revealed a significant loss of neurons in the motor nuclei Mo5 and 7N accumulating intracellular Aβ, while no reductions in cholinergic neuron numbers were detected in other regions of the cholinergic system, such as the forebrain or pons complexes. Importantly, these areas are devoid of human APP expression and Aβ accumulation [54].

## 5. Conclusions

Though there is not a single mouse model mimicking all relevant aspects of AD, nowadays, a huge “toolbox” of models is available that reflect the major AD hallmarks, such as extracellular Aβ deposition, Tau hyperphosphorylation and aspects of neurodegeneration. As outlined in the present review, neuron loss is a valid characteristic in a variety of single and multiple transgenic lines and especially pyramidal neuron loss appears related to intraneuronal Aβ accumulation. The importance of this concept for sporadic AD is, however, still unclear and the obvious transient nature of these accumulations complicate studies in human post-mortem material. If, as in many of the described transgenic models, intraneuronal Aβ accumulation represents a hallmark that precedes overt extracellular plaque deposition and neuron loss, it might be only detectable in an early disease state and its relevance might be underestimated. While data from human studies is scarce, few reports from the human AD brain [13] or young individuals with Down syndrome [138] support such an assumption.

The availability of mouse models reflecting the predominant sporadic form of AD is an important aspect of current research efforts. While novel *APP* knock-in models, such as APP^NL^*,* APP^NL-F^, or APP^NL-G-F^ mice overproduce Aβ_1–42_ in the absence of potentially confounding *APP* overexpression [139], these mice still contain one or more independent FAD mutations, which might result in an abnormal Aβ conformation [140]. The investigation of the contribution of major risk factors associated with SAD is also important. The ε4 variant of the apolipoprotein receptor E (*APOE*) gene is associated with a several-fold increased AD risk and seems to influence brain Aβ clearance and aggregation [141]. It has also been linked to intraneuronal Aβ accumulation in the human AD brain [142] and its fragments seem to promote cellular Aβ uptake [143]. While it has not been demonstrated yet that ApoE variants impact neuron loss in APP transgenic mice, an exacerbation of the neurodegenerative phenotype has been reported in a Tau transgenic model bred on a human ApoE4 genetic background [144]. Another, more recently identified risk factor is the triggering receptor expressed on myeloid cells 2 (*TREM2*), where genetic variants also increase SAD risk [145]. Trem2 deficiency attenuates neurodegeneration in Tau transgenic mice and seems to reduce the neuroinflammatory response [146]. In APP transgenic mice, Aβ plaques in Trem2-deficient 5XFAD mice were more diffuse and associated with more neuritic damage [147]; however, information on overt neurodegeneration is currently lacking. Future studies are needed to explore whether modifications of these SAD risk factors also influence neuron loss in APP/Aβ-driven AD mouse models.

## Figures and Tables

**Table 1:** Transgenic Mouse Models of Alzheimer’s Disease (AD) with Neuron Loss.

Mouse Model	Gene^Mutation^/ Transgene	Promoter	Pathology			Behavior Deficits	Reference
			Aβ Plaques	Intraneuronal Aβ	Neuron Loss		
**Single Transgenic Mouse Models of AD Overexpressing Human APP**							
**Tg2576**	APP^K670N/M671L^	PrP	Yes	Yes	Yes: PN	Yes	[38]
**TgCNRD8**	APP^K670N/M671L,V717F^	Hamster PrP	Yes	NR	Yes: PN, IN	Yes	[39]
**APP23**	APP^K670N/M671L^	Thy1	Yes	Yes	Yes: PN	Yes	[40,41]
**APP_E693Δ_**	APP^E693Δ^	PrP	No	Yes	Yes: PN	Yes	[42]
**TgSweDI**	APP^K670N/M671L,E693Q,D694N^	Thy1	Yes	NR	Yes: CN	Yes	[43,44]
**APP_Swe/Ld_**	APP^K670N/M671L,V717I^	Thy1	Yes	Yes	Yes: CN	Yes	[45]
**Double and Triple Transgenic Mouse Models of AD Overexpressing Human APP**							
**5xFAD**	APP^K670N/M671L,I716V,V717I^ PS1^M146L,L286V^	Thy1	Yes	Yes	Yes: PN, IN, CN	Yes	[46,47,48]
**APP/PS1**	APP^K670N/M671L^ PS1^M146L^	Thy1 HMG-CoA	Yes	Yes	Yes: PN	Yes	[49,50,51]
**APP/PS1KI**	APP^K670N/M671L,V717I^ PS1^M233T/L235P^ KI	Thy1	Yes	Yes	Yes: PN, IN, CN	Yes	[52,53,54]
**APP/Tau**	APP^K670N/M671L^ MAPT^P301L^	PrP Thy1	Yes	Yes	Yes: PN	Yes	[55]
**5xFAD/PS19**	APP^K670N/M671L,I716V,V717I^ PS1^M146L,L286V^ MAPT^P301S^	Thy1 PrP	Yes	Yes	Yes: PN	Yes	[56,57]
**3xTg**	APP^K670N/M671L^ MAPT^P301L^ PS1^M146V^ KI	Thy1	Yes	Yes	Yes: IN, CN	Yes	[58,59]
**5xFAD/Tg30**	APP^K670N/M671L,I716V,V717I^ PS1^M146L,L286V^ Tau(1N4R)^P301S,G272V^	Thy1	Yes	Yes	Yes: PN	Yes	[60]
**Transgenic Mouse Models of AD Overexpressing Human Aβ**							
**NF_L_-Aβ**	murine Aβ	NF_L_	Scarce	Yes	Yes: PN	NR	[61]
**APP48**	rSPENK-Aβ_1–42_	Thy1	No	Yes	Yes: PN	NR	[62]
**TBA2**	mTRH-Aβ_3Q–42_	Thy1	No	Yes	Yes: PN	Yes	[63]
**TBA42**	mTRH-Aβ_3Q–42_	Thy1	No	Yes	Yes: PN	Yes	[64]
**Tg4-42**	mTRH-Aβ_4–42_	Thy1	No	Yes	Yes: PN	Yes	[65]

Abbreviations: Aβ: Amyloid-β, APP: Amyloid precursor protein, AD: Alzheimer’s disease, CN: cholinergic neurons, h: human, IN: interneurons, m: murin, NF_L_: neurofilament light, NR: not reported, PDGF: platelet-derived growth factor, PN: pyramidal neurons, PrP: prion receptor protein, PS1/2: presenilin 1/2, r: rat, SPENK: preproenkephalin signal peptide, TRH: thyrotropin releasing hormone.

## Abbreviations

APP	Amyloid precursor protein
Aβ	Amyloid-β
AD	Alzheimer’s disease
APOE	Apolipoprotein E
BACE1	β-site APP cleaving enzyme 1
ChAT	Choline acetyltransferase
CR	Calretinin
ER	Endoplasmic reticulum
FAD	Familial Alzheimer’s disease
HEK	Human embryonic kidney
KI	Knock-in
nbM	Nucleus basalis of Meynert
NF_L_	Neurofilament light
PV	Parvalbumin
PS	Presenilin
SAD	Sporadic Alzheimer’s disease
TREM2	Triggering receptor expressed on myeloid cells 2

## References

[1] DeTure M.A., Dickson D.W. (2019). The neuropathological diagnosis of Alzheimer’s disease. Mol. Neurodegener..

[2] Braak H., Braak E. (1991). Neuropathological stageing of Alzheimer-related changes. Acta Neuropathol..

[3] Sinha S., Anderson J.P., Barbour R., Basi G.S., Caccavello R., Davis D., Doan M., Dovey H.F., Frigon N., Hong J. (1999). Purification and cloning of amyloid precursor protein beta-secretase from human brain. Nature.

[4] Vassar R., Bennett B.D., Babu-Khan S., Kahn S., Mendiaz E.A., Denis P., Teplow D.B., Ross S., Amarante P., Loeloff R. (1999). Beta-Secretase Cleavage of Alzheimer’s Amyloid Precursor Protein by the Transmembrane Aspartic Protease BACE. Science.

[5] Selkoe D.J., Wolfe M.S. (2007). Presenilin: Running with Scissors in the Membrane. Cell.

[6] Thinakaran G., Koo E.H. (2008). Amyloid Precursor Protein Trafficking, Processing, and Function. J. Biol. Chem..

[7] Klafki H.-W., Rieper P., Matzen A., Zampar S., Wirths O., Vogelgsang J., Osterloh D., Rohdenburg L., Oberstein T.J., Jahn O. (2020). Development and Technical Validation of an Immunoassay for the Detection of APP_669–711_ (Aβ_−3–40_) in Biological Samples. Int. J. Mol. Sci..

[8] Steiner H., Fukumori A., Tagami S., Okochi M. (2018). Making the final cut: Pathogenic amyloid-β peptide generation by γ-secretase. Cell Stress.

[9] Hardy J. (2009). The amyloid hypothesis for Alzheimer’s disease: A critical reappraisal. J. Neurochem..

[10] Hardy J., Allsop D. (1991). Amyloid deposition as the central event in the aetiology of Alzheimer’s disease. Trends Pharmacol. Sci..

[11] Giannakopoulos P., Herrmann F.R., Bussiere T., Bouras C., Kovari E., Perl D.P., Morrison J.H., Gold G., Hof P.R. (2003). Tangle and neuron numbers, but not amyloid load, predict cognitive status in Alzheimer’s disease. Neurology.

[12] Wirths O., Multhaup G., Bayer T.A. (2004). A modified beta-amyloid hypothesis: Intraneuronal accumulation of the beta-amyloid peptide—The first step of a fatal cascade. J. Neurochem..

[13] Gouras G.K., Tsai J., Naslund J., Vincent B., Edgar M., Checler F., Greenfield J.P., Haroutunian V., Buxbaum J.D., Xu H. (2000). Intraneuronal Abeta42 accumulation in human brain. Am. J. Pathol..

[14] Mori C., Spooner E.T., E Wisniewsk K., Wisniewski T., Yamaguch H., Saido T.C., Tolan D.R., Selkoe D.J., A Lemere C. (2002). Intraneuronal Abeta42 accumulation in Down syndrome brain. Amyloid.

[15] D’Andrea M.R., Nagele R.G., Wang H.Y., A Peterson P., Lee D.H.S. (2001). Evidence that neurones accumulating amyloid can undergo lysis to form amyloid plaques in Alzheimer’s disease. Histopathology.

[16] Gouras G.K., Almeida C.G., Takahashi R.H. (2005). Intraneuronal Abeta accumulation and origin of plaques in Alzheimer’s disease. Neurobiol. Aging.

[17] Wirths O., Zampar S. (2019). Emerging roles of N- and C-terminally truncated Aβ species in Alzheimer’s disease. Expert Opin. Ther. Targets.

[18] Coleman P.D., Flood D.G. (1987). Neuron numbers and dendritic extent in normal aging and Alzheimer’s disease. Neurobiol. Aging.

[19] Hof P.R., Morrison J.H., Cox K. (1990). Quantitative analysis of a vulnerable subset of pyramidal neurons in Alzheimer’s disease: I. Superior frontal and inferior temporal cortex. J. Comp. Neurol..

[20] Gómez-Isla T., Price J.L., McKeel D.W., Morris J.C., Growdon J.H., Hyman B.T. (1996). Profound Loss of Layer II Entorhinal Cortex Neurons Occurs in Very Mild Alzheimer’s Disease. J. Neurosci..

[21] Mountjoy C., Roth M., Evans N., Evans H. (1983). Cortical neuronal counts in normal elderly controls and demented patients. Neurobiol. Aging.

[22] Simić G., Kostović I., Winblad B., Bogdanovic N. (1997). Volume and number of neurons of the human hippocampal formation in normal aging and Alzheimer’s disease. J. Comp. Neurol..

[23] West M., Coleman P., Flood D., Troncoso J. (1994). Differences in the pattern of hippocampal neuronal loss in normal ageing and Alzheimer’s disease. Lancet.

[24] Mukhin V.N., Pavlov K.I., Klimenko V.M. (2017). Mechanisms of Neuron Loss in Alzheimer’s Disease. Neurosci. Behav. Physiol..

[25] Bancher C., Lassmann H., Breitschopf H., Jellinger K.A. (1997). Mechanisms of cell death in Alzheimer’s disease. Advances in Research on Neurodegeneration.

[26] Liao D., Miller E.C., Teravskis P.J. (2014). Tau acts as a mediator for Alzheimer’s disease-related synaptic deficits. Eur. J. Neurosci..

[27] Adalbert R., Nogradi A., Babetto E., Janeckova L., Walker S.A., Kerschensteiner M., Misgeld T., Coleman M.P. (2008). Severely dystrophic axons at amyloid plaques remain continuous and connected to viable cell bodies. Brain.

[28] Stokin G.B., Lillo C., Falzone T.L., Brusch R.G., Rockenstein E., Mount S.L., Raman R., Davies P., Masliah E., Williams D.S. (2005). Axonopathy and Transport Deficits Early in the Pathogenesis of Alzheimer’s Disease. Science.

[29] Wirths O., Weis J., Kayed R., Saido T.C., Bayer T.A. (2007). Age-dependent axonal degeneration in an Alzheimer mouse model. Neurobiol. Aging.

[30] Wirths O., Weis J., Szczygielski J., Multhaup G., Bayer T.A. (2006). Axonopathy in an APP/PS1 transgenic mouse model of Alzheimer’s disease. Acta Neuropathol..

[31] Coleman M.P. (2005). Axon degeneration mechanisms: Commonality amid diversity. Nat. Rev. Neurosci..

[32] Roth M., Tomlinson B.E., Blessed G. (1966). Correlation between Scores for Dementia and Counts of ‘Senile Plaques’ in Cerebral Grey Matter of Elderly Subjects. Nat. Cell Biol..

[33] Regeur L., Jensen G.B., Pakkenberg H., Evans S., Pakkenberg B. (1994). No global neocortical nerve cell loss in brains from patients with senile dementia of Alzheimer’s type. Neurobiol. Aging.

[34] Terry R.D., Masliah E., Salmon D.P., Butters N., Bs R.D., Hill R., Hansen L.A., Katzman R. (1991). Physical basis of cognitive alterations in alzheimer’s disease: Synapse loss is the major correlate of cognitive impairment. Ann. Neurol..

[35] Wegiel J., Wisniewski H.M., Tarnawski M., Bobinski M., Reisberg B., De Leon M.J., Miller D.C. (1996). Neurofibrillary pathology—Correlation with hippocampal formation atrophy in Alzheimer disease. Neurobiol. Aging.

[36] Urbanc B., Cruz L., Le R., Sanders J., Ashe K.H., Duff K., Stanley H.E., Irizarry M.C., Hyman B.T. (2002). Neurotoxic effects of thioflavin S-positive amyloid deposits in transgenic mice and Alzheimer’s disease. Proc. Natl. Acad. Sci. USA.

[37] Xie H., Hou S., Jiang J., Sekutowicz M., Kelly J., Bacskai B.J. (2013). Rapid cell death is preceded by amyloid plaque-mediated oxidative stress. Proc. Natl. Acad. Sci. USA.

[38] Hsiao K., Chapman P., Nilsen S., Eckman C., Harigaya Y., Younkin S., Yang F., Cole G. (1996). Correlative memory deficits, Abeta elevation, and amyloid plaques in transgenic mice. Science.

[39] Chishti M.A., Yang D.-S., Janus C., Phinney A.L., Horne P., Pearson J., Strome R., Zuker N., Loukides J., French J. (2001). Early-onset Amyloid Deposition and Cognitive Deficits in Transgenic Mice Expressing a Double Mutant Form of Amyloid Precursor Protein 695. J. Biol. Chem..

[40] Sturchler-Pierrat C., Abramowski D., Duke M., Wiederhold K.-H., Mistl C., Rothacher S., Ledermann B., Bürki K., Frey P., Paganetti P.A. (1997). Two amyloid precursor protein transgenic mouse models with Alzheimer disease-like pathology. Proc. Natl. Acad. Sci. USA.

[41] Calhoun M.E., Wiederhold K.-H., Abramowski R., Phinney A.L., Probst A., Sturchler-Pierrat C., Staufenbiel M., Sommer B., Jucker M. (1998). Neuron loss in APP transgenic mice. Nat. Cell Biol..

[42] Tomiyama T., Matsuyama S., Iso H., Umeda T., Takuma H., Ohnishi K., Ishibashi K., Teraoka R., Sakama N., Yamashita T. (2010). A mouse model of amyloid-b oligomers: Their contribution to synaptic alteration, abnormal tau phosphorylation, glial activation, and neuronal loss in vivo. J Neurosci..

[43] Davis J., Xu F., Deane R., Romanov G., Previti M.L., Zeigler K., Zlokovic B.V., Van Nostrand W.E. (2004). Early-onset and Robust Cerebral Microvascular Accumulation of Amyloid β-Protein in Transgenic Mice Expressing Low Levels of a Vasculotropic Dutch/Iowa Mutant Form of Amyloid β-Protein Precursor. J. Biol. Chem..

[44] Foidl B.M., Do-Dinh P., Hutter-Schmid B., Bliem H.R., Humpel C. (2016). Cholinergic neurodegeneration in an Alzheimer mouse model overexpressing amyloid-precursor protein with the Swedish-Dutch-Iowa mutations. Neurobiol. Learn. Mem..

[45] Ubhi K., Rockenstein E., Vazquez-Roque R., Mante M., Inglis C., Patrick C., Adame A., Fahnestock M., Doppler E., Novak P. (2012). Cerebrolysin modulates pronerve growth factor/nerve growth factor ratio and ameliorates the cholinergic deficit in a transgenic model of Alzheimer’s disease. J. Neurosci. Res..

[46] Oakley H., Cole S.L., Logan S., Maus E., Shao P., Craft J., Guillozet-Bongaarts A., Ohno M., Disterhoft J., Van Eldik L. (2006). Intraneuronal beta-Amyloid Aggregates, Neurodegeneration, and Neuron Loss in Transgenic Mice with Five Familial Alzheimer’s Disease Mutations: Potential Factors in Amyloid Plaque Formation. J. Neurosci..

[47] Eimer W.A., Vassar R. (2013). Neuron loss in the 5XFAD mouse model of Alzheimer’s disease correlates with intraneuronal Abeta42 accumulation and Caspase-3 activation. Mol. Neurodegener..

[48] Jawhar S., Trawicka A., Jenneckens C., Bayer T.A., Wirths O. (2012). Motor deficits, neuron loss, and reduced anxiety coinciding with axonal degeneration and intraneuronal Abeta aggregation in the 5XFAD mouse model of Alzheimer’s disease. Neurobiol. Aging.

[49] Blanchard V., Moussaoui S., Czech C., Touchet N., Bonici B., Planche M., Canton T., Jedidi I., Gohin M., Wirths O. (2003). Time sequence of maturation of dystrophic neurites associated with Abeta deposits in APP/PS1 transgenic mice. Exp. Neurol..

[50] Schmitz C., Rutten B.P.F., Pielen A., Schäfer S., Wirths O., Tremp G., Czech C., Blanchard V., Multhaup G., Rezaie P. (2004). Hippocampal Neuron Loss Exceeds Amyloid Plaque Load in a Transgenic Mouse Model of Alzheimer’s Disease. Am. J. Pathol..

[51] Wirths O., Multhaup G., Czech C., Feldmann N., Blanchard V., Tremp G., Beyreuther K., Pradier L., Bayer T.A. (2002). Intraneuronal APP/A beta trafficking and plaque formation in beta-amyloid precursor protein and presenilin-1 transgenic mice. Brain Pathol..

[52] Casas C., Sergeant N., Itier J.M., Blanchard V., Wirths O., van der Kolk N., Vingtdeux V., van de Steeg E., Ret G., Canton T. (2004). Massive CA1/2 neuronal loss with intraneuronal and N-terminal truncated Abeta42 accumulation in a novel Alzheimer transgenic model. Am. J. Pathol..

[53] Christensen D.Z., Kraus S.L., Flohr A., Cotel M.C., Wirths O., Bayer T.A. (2008). Transient intraneuronal Abeta rather than extracellular plaque pathology correlates with neuron loss in the frontal cortex of APP/PS1KI mice. Acta Neuropathol..

[54] Christensen D.Z., Bayer T.A., Wirths O. (2010). Intracellular Aβ triggers neuron loss in the cholinergic system of the APP/PS1KI mouse model of Alzheimer’s disease. Neurobiol. Aging.

[55] Ribe E.M., Perez M., Puig B., Gich I., Lim F., Cuadrado-Tejedor M., Sesma T., Catena S., Sanchez B.U., Nieto M. (2005). Accelerated amyloid deposition, neurofibrillary degeneration and neuronal loss in double mutant APP/tau transgenic mice. Neurobiol. Dis..

[56] Saul A., Sprenger F., Bayer T.A., Wirths O. (2013). Accelerated tau pathology with synaptic and neuronal loss in a novel triple transgenic mouse model of Alzheimer’s disease. Neurobiol. Aging.

[57] Stancu I., Ris L., De Vasconcelos B.B., Marinangeli C., Goeminne L., Laporte V., Haylani L.E., Couturier J., Schakman O., Gailly P. (2014). Tauopathy contributes to synaptic and cognitive deficits in a murine model for Alzheimer’s disease. FASEB J..

[58] Girao da Cruz M.T., Jordao J., Dasilva K.A., Ayala-Grosso C.A., Ypsilanti A., Weng Y.Q., Laferla F.M., McLaurin J., Aubert I. (2012). Early Increases in Soluble Amyloid-beta Levels Coincide with Cholinergic Degeneration in 3xTg-AD mice. J. Alzheimers Dis..

[59] Zallo F., Gardenal E., Verkhratsky A., Rodríguez J.J. (2018). Loss of calretinin and parvalbumin positive interneurones in the hippocampal CA1 of aged Alzheimer’s disease mice. Neurosci. Lett..

[60] Héraud C., Goufak D., Ando K., Leroy K., Suain V., Yilmaz Z., De Decker R., Authelet M., Laporte V., Octave J.-N. (2014). Increased misfolding and truncation of tau in APP/PS1/tau transgenic mice compared to mutant tau mice. Neurobiol. Dis..

[61] LaFerla F.M., Tinkle B.T., Bieberich C.J., Haudenschild C.C., Jay G. (1995). The Alzheimer’s A beta peptide induces neurodegeneration and apoptotic cell death in transgenic mice. Nat. Genet..

[62] Abramowski D., Rabe S., Upadhaya A.R., Reichwald J., Danner S., Staab D., Capetillo-Zarate E., Yamaguchi H., Saido T.C., Wiederhold K.-H. (2012). Transgenic Expression of Intraneuronal Aβ42 But Not Aβ40 Leads to Cellular Aβ Lesions, Degeneration, and Functional Impairment without Typical Alzheimer’s Disease Pathology. J. Neurosci..

[63] Wirths O., Breyhan H., Cynis H., Schilling S., DeMuth H.-U., Bayer T.A. (2009). Intraneuronal pyroglutamate-Abeta 3–42 triggers neurodegeneration and lethal neurological deficits in a transgenic mouse model. Acta Neuropathol..

[64] Wittnam J.L., Portelius E., Zetterberg H., Gustavsson M.K., Schilling S., Koch B., DeMuth H.-U., Blennow K., Wirths O., Bayer T.A. (2012). Pyroglutamate Amyloid β (Aβ) Aggravates Behavioral Deficits in Transgenic Amyloid Mouse Model for Alzheimer Disease. J. Biol. Chem..

[65] Bouter Y., Dietrich K., Wittnam J.L., Rezaei-Ghaleh N., Pillot T., Papot-Couturier S., Lefebvre T., Sprenger F., Wirths O., Zweckstetter M. (2013). N-truncated amyloid β (Aβ) 4-42 forms stable aggregates and induces acute and long-lasting behavioral deficits. Acta Neuropathol..

[66] Irizarry M.C., McNamara M., Fedorchak K., Hsiao K., Hyman B.T. (1997). APPSw transgenic mice develop age-related A beta deposits and neuropil abnormalities, but no neuronal loss in CA1. J. Neuropathol. Exp. Neurol..

[67] Irizarry M.C., Soriano F., McNamara M., Page K.J., Schenk D.B., Games D., Hyman B.T. (1997). Aβ Deposition Is Associated with Neuropil Changes, but not with Overt Neuronal Loss in the Human Amyloid Precursor Protein V717F (PDAPP) Transgenic Mouse. J. Neurosci..

[68] Games D., Adams D., Alessandrini R., Barbour R., Berthelette P., Blackwell C., Carr T., Clemens J., Donaldson T., Gillespie F. (1995). Alzheimer-type neuropathology in transgenic mice overexpressing V717F beta-amyloid precursor protein. Nature.

[69] Moechars D., Dewachter I., Lorent K., Reversé D., Baekelandt V., Naidu A., Tesseur I., Spittaels K., Haute C.V.D., Checler F. (1999). Early Phenotypic Changes in Transgenic Mice That Overexpress Different Mutants of Amyloid Precursor Protein in Brain. J. Biol. Chem..

[70] Lord A., Kalimo H., Eckman C., Zhang X.Q., Lannfelt L., Nilsson L.N. (2006). The Arctic Alzheimer mutation facilitates early intraneuronal Abeta aggregation and senile plaque formation in transgenic mice. Neurobiol. Aging.

[71] Van Broeck B., Vanhoutte G., Pirici D., Van Dam D., Wils H., Cuijt I., Vennekens K., Zabielski M., Michalik A., Theuns J. (2008). Intraneuronal amyloid β and reduced brain volume in a novel APP T714I mouse model for Alzheimer’s disease. Neurobiol. Aging.

[72] Borchelt D.R., Ratovitski T., Van Lare J., Lee M.K., Gonzales V., A Jenkins N., Copeland N.G., Price D.L., Sisodia S.S. (1997). Accelerated Amyloid Deposition in the Brains of Transgenic Mice Coexpressing Mutant Presenilin 1 and Amyloid Precursor Proteins. Neuron.

[73] Richards G., Higgins G.A., Ouagazzal A.-M., Ozmen L., Kew J.N.C., Bohrmann B., Malherbe P., Brockhaus M., Loetscher H., Czech C. (2003). PS2APP Transgenic Mice, Coexpressing hPS2mut and hAPPswe, Show Age-Related Cognitive Deficits Associated with Discrete Brain Amyloid Deposition and Inflammation. J. Neurosci..

[74] Oddo S., Caccamo A., Shepherd J.D., Murphy M., Golde T.E., Kayed R., Metherate R., Mattson M.P., Akbari Y., LaFerla F.M. (2003). Triple-Transgenic Model of Alzheimer’s Disease with Plaques and Tangles. Neuron.

[75] Grueninger F., Bohrmann B., Czech C., Ballard T.M., Frey J.R., Weidensteiner C., Von Kienlin M., Ozmen L. (2010). Phosphorylation of Tau at S422 is enhanced by Aβ in TauPS2APP triple transgenic mice. Neurobiol. Dis..

[76] Bondolfi L., Calhoun M., Ermini F., Kuhn H.G., Wiederhold K.-H., Walker L.C., Staufenbiel M., Jucker M. (2002). Amyloid-Associated Neuron Loss and Gliogenesis in the Neocortex of Amyloid Precursor Protein Transgenic Mice. J. Neurosci..

[77] Rijal Upadhaya A., Scheibe F., Kosterin I., Abramowski D., Gerth J., Kumar S., Liebau S., Yamaguchi H., Walter J., Staufenbiel M. (2013). The type of Abeta-related neuronal degeneration differs between amyloid precursor protein (APP23) and amyloid beta-peptide (APP48) transgenic mice. Acta Neuropathol. Commun..

[78] Wegenast-Braun B.M., Maisch A.F., Eicke D., Radde R., Herzig M.C., Staufenbiel M., Jucker M., Calhoun M.E. (2009). Independent Effects of Intra- and Extracellular Aβ on Learning-Related Gene Expression. Am. J. Pathol..

[79] Langui D., Girardot N., El Hachimi K.H., Allinquant B., Blanchard V., Pradier L., Duyckaerts C. (2004). Subcellular Topography of Neuronal Aβ Peptide in APPxPS1 Transgenic Mice. Am. J. Pathol..

[80] Takahashi R.H., Milner T.A., Li F., Nam E.E., Edgar M.A., Yamaguchi H., Beal M.F., Xu H., Greengard P., Gouras G.K. (2002). Intraneuronal Alzheimer Aβ42 Accumulates in Multivesicular Bodies and Is Associated with Synaptic Pathology. Am. J. Pathol..

[81] Rutten B.P.F., Van Der Kolk N.M., Schäfer S., Van Zandvoort M.A., Bayer T.A., Steinbusch H.W., Schmitz C. (2005). Age-Related Loss of Synaptophysin Immunoreactive Presynaptic Boutons within the Hippocampus of APP751SL, PS1M146L, and APP751SL/PS1M146L Transgenic Mice. Am. J. Pathol..

[82] Breyhan H., Wirths O., Duan K., Marcello A., Rettig J., Bayer T.A. (2009). APP/PS1KI bigenic mice develop early synaptic deficits and hippocampus atrophy. Acta Neuropathol..

[83] Cotel M.-C., Bayer T.A., Wirths O. (2008). Age-dependent loss of dentate gyrus granule cells in APP/PS1KI mice. Brain Res..

[84] Ohno M., Cole S.L., Yasvoina M., Zhao J., Citron M., Berry R., Disterhoft J.F., Vassar R. (2007). BACE1 gene deletion prevents neuron loss and memory deficits in 5XFAD APP/PS1 transgenic mice. Neurobiol. Dis..

[85] Leroy K., Ando K., Laporte V., Dedecker R., Suain V., Authelet M., Héraud C., Pierrot N., Yilmaz Z., Octave J.-N. (2012). Lack of Tau Proteins Rescues Neuronal Cell Death and Decreases Amyloidogenic Processing of APP in APP/PS1 Mice. Am. J. Pathol..

[86] Tomiyama T., Nagata T., Shimada H., Teraoka R., Fukushima A., Kanemitsu H., Takuma H., Kuwano R., Imagawa M., Ataka S. (2008). A new amyloid beta variant favoring oligomerization in Alzheimer’s-type dementia. Ann. Neurol..

[87] Benilova I., Karran E., De Strooper B. (2012). The toxic Abeta oligomer and Alzheimer’s disease: An emperor in need of clothes. Nat. Neurosci..

[88] Walsh D.M., Selkoe D.J. (2007). Aβ Oligomers–a decade of discovery. J. Neurochem..

[89] Nishitsuji K., Tomiyama T., Ishibashi K., Ito K., Teraoka R., Lambert M.P., Klein W.L., Mori H. (2009). The E693{Delta} Mutation in Amyloid Precursor Protein Increases Intracellular Accumulation of Amyloid {beta} Oligomers and Causes Endoplasmic Reticulum Stress-Induced Apoptosis in Cultured Cells. Am. J Pathol..

[90] Umeda T., Tomiyama T., Sakama N., Tanaka S., Lambert M., Klein W., Mori H. (2011). P3-134: Intraneuronal amyloid-beta oligomers cause cell death via endoplasmic reticulum stress, endosomal/lysosomal leakage, and mitochondrial dysfunction in vivo. Alzheimer’s Dement..

[91] Ugolini F., Lana D., Nardiello P., Nosi D., Pantano D., Casamenti F., Giovannini M.G. (2018). Different Patterns of Neurodegeneration and Glia Activation in CA1 and CA3 Hippocampal Regions of TgCRND8 Mice. Front. Aging Neurosci..

[92] Harwell C.S., Coleman M.P. (2016). Synaptophysin depletion and intraneuronal Aβ in organotypic hippocampal slice cultures from huAPP transgenic mice. Mol. Neurodegener..

[93] Comes G., Manso Y., Escrig A., Fernández-Gayol O., Sanchis P., Molinero A., Giralt M., Carrasco J., Hidalgo J. (2017). Influence of Transgenic Metallothionein-1 on Gliosis, CA1 Neuronal Loss, and Brain Metal Levels of the Tg2576 Mouse Model of Alzheimer’s Disease. Int. J. Mol. Sci..

[94] Dunys J., Valverde A., Checler F. (2018). Are N- and C-terminally truncated Aβ species key pathological triggers in Alzheimer’s disease?. J. Biol. Chem..

[95] Saido T., Iwatsubo T., Mann D.M., Shimada H., Ihara Y., Kawashima S. (1995). Dominant and differential deposition of distinct β-amyloid peptide species, AβN3(pE), in senile plaques. Neuron.

[96] Schilling S., Lauber T., Schaupp M., Manhart S., Scheel E., Böhm G., DeMuth H.-U. (2006). On the Seeding and Oligomerization of pGlu-Amyloid Peptides (in vitro). Biochemistry.

[97] Kuo Y.M., Webster S., Emmerling M.R., De Lima N., Roher A.E. (1998). Irreversible dimerization/tetramerization and post-translational modifications inhibit proteolytic degradation of Abeta peptides of Alzheimer’s disease. Biochim. Biophys. Acta.

[98] Alexandru A., Jagla W., Graubner S., Becker A., Bauscher C., Kohlmann S., Sedlmeier R., Raber K.A., Cynis H., Ronicke R. (2011). Selective Hippocampal Neurodegeneration in Transgenic Mice Expressing Small Amounts of Truncated Abeta Is Induced by Pyroglutamate-Abeta Formation. J. Neurosci..

[99] Cynis H., Schilling S., Bodnár M., Hoffmann T., Heiser U., Saido T.C., DeMuth H.-U. (2006). Inhibition of glutaminyl cyclase alters pyroglutamate formation in mammalian cells. Biochim. Biophys. Acta BBA Proteins Proteom..

[100] Meissner J.N., Bouter Y., Bayer T.A. (2015). Neuron Loss and Behavioral Deficits in the TBA42 Mouse Model Expressing N-Truncated Pyroglutamate Amyloid-beta3-42. J. Alzheimers Dis..

[101] Masters C.L., Simms G., Weinman N.A., Multhaup G., McDonald B.L., Beyreuther K. (1985). Amyloid plaque core protein in Alzheimer disease and Down syndrome. Proc. Natl. Acad. Sci. USA.

[102] Portelius E., Bogdanovic N., Gustavsson M.K., Volkmann I., Brinkmalm G., Zetterberg H., Winblad B., Blennow K. (2010). Mass spectrometric characterization of brain amyloid beta isoform signatures in familial and sporadic Alzheimer’s disease. Acta Neuropathol..

[103] Antonios G., Saiepour N., Bouter Y., Richard B.C., Paetau A., Verkkoniemi-Ahola A., Lannfelt L., Ingelsson M., Kovacs G.G., Pillot T. (2013). N-truncated Abeta starting with position four: Early intraneuronal accumulation and rescue of toxicity using NT4X-167, a novel monoclonal antibody. Acta Neuropathol. Commun..

[104] Hüttenrauch M., Brauß A., Kurdakova A., Borgers H., Klinker F., Liebetanz D., Salinas-Riester G., Wiltfang J., Klafki H.W., Wirths O. (2016). Physical activity delays hippocampal neurodegeneration and rescues memory deficits in an Alzheimer disease mouse model. Transl. Psychiatry.

[105] Wagner J.M., Sichler M.E., Schleicher E.M., Franke T.N., Irwin C., Löw M.J., Beindorff N., Bouter C., Bayer T.A., Bouter Y. (2019). Analysis of Motor Function in the Tg4-42 Mouse Model of Alzheimer’s Disease. Front. Behav. Neurosci..

[106] Gerberding A.-L., Zampar S., Stazi M., Liebetanz D., Wirths O. (2019). Physical Activity Ameliorates Impaired Hippocampal Neurogenesis in the Tg4-42 Mouse Model of Alzheimer’s Disease. ASN Neuro.

[107] Lopez-Noguerola J.S., Giessen N.M.E., Ueberück M., Meißner J.N., Pelgrim C.E., Adams J., Wirths O., Bouter Y., Bayer T.A. (2018). Synergistic Effect on Neurodegeneration by N-Truncated Aβ4−42 and Pyroglutamate Aβ3−42 in a Mouse Model of Alzheimer’s Disease. Front. Aging Neurosci..

[108] Mastrangelo M.A., Bowers W.J. (2008). Detailed immunohistochemical characterization of temporal and spatial progression of Alzheimer’s disease-related pathologies in male triple-transgenic mice. BMC Neurosci..

[109] Rohn T.T., Vyas V., Hernandez-Estrada T., Nichol K.E., Christie L.-A., Head E. (2008). Lack of Pathology in a Triple Transgenic Mouse Model of Alzheimer’s Disease after Overexpression of the Anti-Apoptotic Protein Bcl-2. J. Neurosci..

[110] Manaye K.F., Mouton P.R., Xu G., Drew A., Lei D.-L., Sharma Y., Rebeck G.W., Turner R.S. (2011). Age-related loss of noradrenergic neurons in the brains of triple transgenic mice. AGE.

[111] Aho L., Pikkarainen M., Hiltunen M., Leinonen V., Alafuzoff I. (2010). Immunohistochemical Visualization of Amyloid-β Protein Precursor and Amyloid-β in Extra- and Intracellular Compartments in the Human Brain. J. Alzheimer’s Dis..

[112] Wirths O., Dins A., Bayer T.A. (2012). AbetaPP Accumulation and/or Intraneuronal Amyloid-beta Accumulation? The 3xTg-AD Mouse Model Revisited. J. Alzheimers Dis..

[113] Yoshiyama Y., Higuchi M., Zhang B., Huang S.-M., Iwata N., Saido T.C., Maeda J., Suhara T., Trojanowski J.Q., Lee V.M.-Y. (2007). Synapse Loss and Microglial Activation Precede Tangles in a P301S Tauopathy Mouse Model. Neuron.

[114] Bloom G.S. (2014). Amyloid-β and tau: The trigger and bullet in alzheimer disease pathogenesis. JAMA Neurol..

[115] Rapoport M., Dawson H.N., Binder L.I., Vitek M.P., Ferreira A. (2002). Tau is essential to beta -amyloid-induced neurotoxicity. Proc. Natl. Acad. Sci. USA.

[116] Stancu I.-C., Vasconcelos B., Terwel D., Dewachter I. (2014). Models of β-amyloid induced Tau-pathology: The long and “folded” road to understand the mechanism. Mol. Neurodegener..

[117] Pelkey K.A., Chittajallu R., Craig M.T., Tricoire L., Wester J.C., McBain C.J. (2017). Hippocampal GABAergic Inhibitory Interneurons. Physiol. Rev..

[118] Brady D., Mufson E. (1997). Parvalbumin-immunoreactive neurons in the hippocampal formation of Alzheimer’s diseased brain. Neuroscience.

[119] Sanchez-Mejias E., Nuñez-Diaz C., Sanchez-Varo R., Gomez-Arboledas A., Garcia-Leon J.A., Fernandez-Valenzuela J.J., Mejias-Ortega M., Trujillo-Estrada L., Baglietto-Vargas D., Moreno-Gonzalez I. (2019). Distinct disease-sensitive GABAergic neurons in the perirhinal cortex of Alzheimer’s mice and patients. Brain Pathol..

[120] Mikkonen M., Alafuzoff I., Tapiola T., Soininen H., Miettinen R. (1999). Subfield- and layer-specific changes in parvalbumin, calretinin and calbindin-D28k immunoreactivity in the entorhinal cortex in Alzheimer’s disease. Neuroscience.

[121] Saiz-Sanchez D., De La Rosa-Prieto C., Ubeda-Bañon I., Martinez-Marcos A., Saiz-Sanchez D. (2014). Interneurons, tau and amyloid-β in the piriform cortex in Alzheimer’s disease. Brain Struct. Funct..

[122] Albuquerque M.S., Emahar I., Edavoli M.A., Echabot J.-G., Emechawar N., Quirion R., Krantic S. (2015). Regional and sub-regional differences in hippocampal GABAergic neuronal vulnerability in the TgCRND8 mouse model of Alzheimer’s disease. Front. Aging Neurosci..

[123] Baglietto-Vargas D., Moreno-Gonzalez I., Sanchez-Varo R., Jimenez S., Trujillo-Estrada L., Sanchez-Mejias E., Torres M., Romero-Acebal M., Ruano D., Vizuete M. (2010). Calretinin interneurons are early targets of extracellular amyloid-beta pathology in PS1/AbetaPP Alzheimer mice hippocampus. J. Alzheimers Dis..

[124] Popovic M., Caballero-Bleda M., Kadish I., Van Groen T. (2008). Subfield and layer-specific depletion in calbindin-D28K, calretinin and parvalbumin immunoreactivity in the dentate gyrus of amyloid precursor protein/presenilin 1 transgenic mice. Neuroscience.

[125] Takahashi H., Brasnjevic I., Rutten B.P.F., Van Der Kolk N., Perl D.P., Bouras C., Steinbusch H.W.M., Schmitz C., Hof P.R., Dickstein D.L. (2010). Hippocampal interneuron loss in an APP/PS1 double mutant mouse and in Alzheimer’s disease. Brain Struct. Funct..

[126] Giesers N.K., Wirths O. (2020). Loss of Hippocampal Calretinin and Parvalbumin Interneurons in the 5XFAD Mouse Model of Alzheimer’s Disease. ASN Neuro.

[127] Ali F., Baringer S.L., Neal A., Choi E.Y., Kwan A.C. (2019). Parvalbumin-Positive Neuron Loss and Amyloid-beta Deposits in the Frontal Cortex of Alzheimer’s Disease-Related Mice. J. Alzheimers. Dis..

[128] Flanigan T.J., Xue Y., Rao S.K., Dhanushkodi A., McDonald M.P. (2014). Abnormal vibrissa-related behavior and loss of barrel field inhibitory neurons in 5xFAD transgenics. Genes Brain Behav..

[129] Lemmens M.A.M., Sierksma A.S.R., Rutten B.P.F., Dennissen F.J.A., Steinbusch H.W.M., Lucassen P.J., Schmitz C. (2011). Age-related changes of neuron numbers in the frontal cortex of a transgenic mouse model of Alzheimer’s disease. Brain Struct. Funct..

[130] Mahar I., Albuquerque M.S., Mondragon-Rodriguez S., Cavanagh C., Davoli M.A., Chabot J.-G., Williams S., Mechawar N., Quirion R., Krantic S. (2017). Phenotypic Alterations in Hippocampal NPY- and PV-Expressing Interneurons in a Presymptomatic Transgenic Mouse Model of Alzheimer’s Disease. Front. Aging Neurosci..

[131] Roselli F., Caroni P. (2015). From Intrinsic Firing Properties to Selective Neuronal Vulnerability in Neurodegenerative Diseases. Neuron.

[132] Hampel H., Mesulam M.-M., Cuello A.C., Farlow M.R., Giacobini E., Grossberg G.T., Khachaturian A.S., Vergallo A., Cavedo E., Snyder P.J. (2018). The cholinergic system in the pathophysiology and treatment of Alzheimer’s disease. Brain.

[133] German D., Yazdani U., Speciale S.G., Pasbakhsh P., Games D., Liang C.-L. (2003). Cholinergic neuropathology in a mouse model of Alzheimer’s disease. J. Comp. Neurol..

[134] Boncristiano S., Calhoun M.E., Kelly P.H., Pfeifer M., Bondolfi L., Stalder M., Phinney A.L., Abramowski R., Sturchler-Pierrat C., Enz A. (2002). Cholinergic Changes in the APP23 Transgenic Mouse Model of Cerebral Amyloidosis. J. Neurosci..

[135] Choi J.H., Kaur G., Mazzella M.J., Morales-Corraliza J., Levy E., Mathews P.M. (2013). Early Endosomal Abnormalities and Cholinergic Neuron Degeneration in Amyloid-β Protein Precursor Transgenic Mice. J. Alzheimer’s Dis..

[136] Devi L., Ohno M. (2010). Phospho-eIF2α Level Is Important for Determining Abilities of BACE1 Reduction to Rescue Cholinergic Neurodegeneration and Memory Defects in 5XFAD Mice. PLoS ONE.

[137] Yan H., Pang P., Chen W., Zhu H., K.A. H., Li H., Wu Z., Ke X., Wu J., Zhang T. (2017). The Lesion Analysis of Cholinergic Neurons in 5XFAD Mouse Model in the Three-Dimensional Level of Whole Brain. Mol. Neurobiol..

[138] Gyure K.A., Durham R., Stewart W.F., E Smialek J., Troncoso J.C. (2001). Intraneuronal abeta-amyloid precedes development of amyloid plaques in Down syndrome. Arch. Pathol. Lab. Med..

[139] Saito T., Matsuba Y., Mihira N., Takano J., Nilsson P., Itohara S., Iwata N., Saido T.C. (2014). Single App knock-in mouse models of Alzheimer’s disease. Nat. Neurosci..

[140] Sasaguri H., Nilsson P., Hashimoto S., Nagata K., Saito T., De Strooper B., Hardy J., Vassar R., Winblad B., Saido T.C. (2017). APP mouse models for Alzheimer’s disease preclinical studies. EMBO J..

[141] Holtzman D.M., Herz J., Bu G. (2012). Apolipoprotein E and Apolipoprotein E Receptors: Normal Biology and Roles in Alzheimer Disease. Cold Spring Harb. Perspect. Med..

[142] Christensen D.Z., Schneider-Axmann T., Lucassen P.J., Bayer T.A., Wirths O. (2010). Accumulation of intraneuronal Aβ correlates with ApoE4 genotype. Acta Neuropathol..

[143] Dafnis I., Stratikos E., Tzinia A., Tsilibary E.C., Zannis V.I., Chroni A. (2010). An apolipoprotein E4 fragment can promote intracellular accumulation of amyloid peptide beta 42. J. Neurochem..

[144] Shi Y., Initiative A.D.N., Yamada K., Liddelow S.A., Smith J.A., Zhao L., Luo W., Tsai R.M., Spina S., Grinberg L.T. (2017). ApoE4 markedly exacerbates tau-mediated neurodegeneration in a mouse model of tauopathy. Nat. Cell Biol..

[145] Guerreiro R., Wojtas A., Bras J., Carrasquillo M.M., Rogaeva E., Majounie E., Cruchaga C., Sassi C., Kauwe J.S., Younkin S.G. (2013). TREM2 Variants in Alzheimer’s Disease. N. Engl. J. Med..

[146] Leyns C.E.G., Ulrich J.D., Finn M.B., Stewart F.R., Koscal L.J., Serrano J.R., Robinson G.O., Anderson E., Colonna M., Holtzman D.M. (2017). TREM2 deficiency attenuates neuroinflammation and protects against neurodegeneration in a mouse model of tauopathy. Proc. Natl. Acad. Sci. USA.

[147] Wang Y., Ulland T.K., Ulrich J.D., Song W., Tzaferis J.A., Hole J.T., Yuan P., Mahan T.E., Shi Y., Gilfillan S. (2016). TREM2-mediated early microglial response limits diffusion and toxicity of amyloid plaques. J. Exp. Med..

